# Supplementary material for: Tead1a Initiates Transcriptional Priming Through the TEAD1a/YAP‐Notch1‐Spi1/Cebpα Axis to Promote Neutrophil Fate
Source: Adv Sci (Weinh). 2025 Aug 28;12(41):e05441. doi: 10.1002/advs.202505441 (PMC12591127; doi:10.1002/advs.202505441)
Supplement: Supplementary file 4 — Supplementary Dataset [file ADVS-12-e05441-s004.zip › 3-1.zip!/3-1/02-1.cluster/cluster1/Enrich/ego_all.pdf]

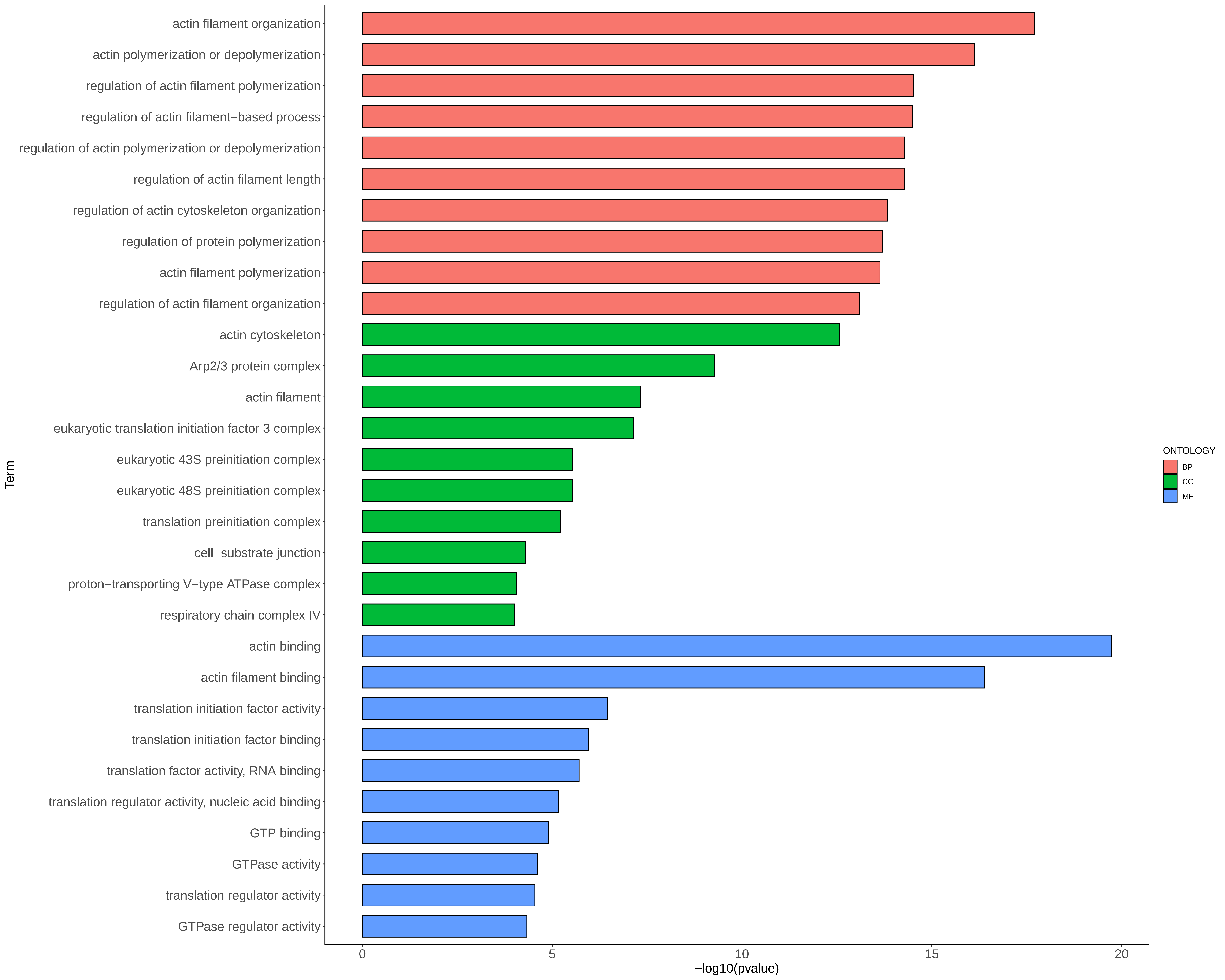
actin filament organization
actin polymerization or depolymerization
regulation of actin filament polymerization
regulation of actin filament−based process
regulation of actin polymerization or depolymerization
regulation of actin filament length
regulation of actin cytoskeleton organization
regulation of protein polymerization
actin filament polymerization
regulation of actin filament organization
actin cytoskeleton
Arp2/3 protein complex
actin filament
eukaryotic translation initiation factor 3 complex
eukaryotic 43S preinitiation complex
eukaryotic 48S preinitiation complex
translation preinitiation complex
cell−substrate junction
proton−transporting V−type ATPase complex
respiratory chain complex IV
actin binding
actin filament binding
translation initiation factor activity
translation initiation factor binding
translation factor activity, RNA binding
translation regulator activity, nucleic acid binding
GTP binding
GTPase activity
translation regulator activity
GTPase regulator activity
Term
0
5
10
15
20
−log10(pvalue)
ONTOLOGY
BP
CC
MF
